# A New Alien Invasive Longhorn Beetle, *Xylotrechus chinensis* (Cerambycidae), Is Infesting Mulberries in Catalonia (Spain)

**DOI:** 10.3390/insects9020052

**Published:** 2018-05-09

**Authors:** Víctor Sarto i Monteys, Glòria Torras i Tutusaus

**Affiliations:** 1 Institut de Ciència i Tecnologia Ambientals (ICTA), Entomology, Plants and Health, Edifici Z–ICTA-ICP Campus de Bellaterra, Universitat Autònoma de Barcelona, 08193 Bellaterra, Spain; 2 Servei de Sanitat Vegetal, DARP, Generalitat de Catalunya, Av. Meridiana, 38, 08018 Barcelona, Spain; 3 Ajuntament de Barberà del Vallès, Serveis Territorials, Parcs i Jardins. Circumval·lació, 14, 08210 Barberà del Vallès, Spain; torrastg@bdv.cat

**Keywords:** invasive longhorn beetle, Cerambycidae, *Xylotrechus chinensis*, mulberry trees damage, *Morus* sp. pest, common grape vine threat, *Vitis vinifera*

## Abstract

In this paper, the invasion of a new alien beetle species to Europe, the longhorn *Xylotrechus chinensis* (Chevrolat) (Cerambycidae), originating from East Asia, is revealed. It has settled in Catalonia (Spain), occupying at present an area of at least 44.1 km^2^, where it has been shown to severely infest (ca. 10 to 45%) and eventually kill mulberry trees in private and public grounds. The main objective of this study was to evaluate its impact and provide new significant insights into its life history, seasonality, reproductive capacity (females produce an average of 83.4 ± 9.02 eggs) and the type of damage produced to mulberries. Such damage was thoroughly described to facilitate inspection by others. At least in laboratory conditions, *X. chinensis* has not used common grape vines as an alternative hostplant. Both plants, mulberries and grape vines, are important in Catalonia and Spain, the former providing shade and ornament to many streets and avenues, and the latter having great economic significance in Mediterranean wine production areas. Possible control methods to hinder its spread are suggested and one local wasp, *Stephanus serrator* (Stephanidae), was identified as a likely parasitoid. We believe the risk of this beetle widely spreading in Europe is very real.

## 1. Introduction

Cerambycids or longhorn beetles (Cerambycidae) play critical roles in nutrient cycling in forests since larval feeding initiates the breakdown of woody tissues while simultaneously creating access routes for wood-rotting fungi and other wood-boring agents [1]. Their larvae prefer boring in stressed, dying, or recently-dead woody plants but might also bore in mature, healthy trees if the other group is not available [2].

Host plant odours attract both sexes of many cerambycid species and, once on the host, males locate females and recognise them by the contact chemoreception of female-produced scents [3]. Volatile attractant pheromones also play a role in mate location in cerambycid species, although in Cerambycinae, only male-produced aggregation-sex pheromones, that attract both sexes, are known (see [4] for review).

Some cerambycids are invasive in other territories. When introduced into new regions of the world, exotic invasive cerambycids have the potential to become devastating pests since there exists great difficulty and cost in detecting, controlling, or eradicating them from invaded regions [4]. A total of 19 exotic invasive longhorn beetle species (9 Cerambycinae, 7 Lamiinae, 2 Prioninae and 1 Parandrinae) have established in Europe, mainly during the period 1975–1999 and arriving predominantly from Asia [5]. They were all introduced accidentally, with France, Spain and Italy the most frequently invaded countries. Wood-derived products such as wood-packaging material and pallets, plants for planting, and bonsais constitute invasive pathways of increasing importance for longhorn beetles. To date in Europe, few species have colonised natural habitats outside parks and gardens [5].

The genus *Xylotrechus* Chevrolat 1860 (Cerambycinae, tribe Clytini) is Holarctic in origin and richest in species diversity in the Eastern Palaearctic [6]. At least 180 *Xylotrechus* species are recorded worldwide [7] with around 30 species in North America [8,9,10] and 85 in the Palaearctic [11]. Among the latter are seven European species [12], with four in the Iberian Peninsula: *Xylotrechus rusticus* (Linnaeus 1758), *Xylotrechus arvicola* (Olivier 1795), *Xylotrechus antilope* (Schonherr 1817) and *Xylotrechus stebbingi* Gahan 1906 [13,14]. *X. arvicola* is known to cause damage in Spanish vineyards [15,16].

*Xylotrechus* species, with one exception, are associated with woody plants and their larvae develop in stems and trunks where they feed in the phloem and occasionally the cambium [17]. The exception is *X. arnoldii* Kostin 1974, whose larvae develop in rhizomes of goosefoot herbaceous plants (Amaranthaceae, Chenopodioideae) of steppe fields of Kazakhstan [6]. The genus *Xylotrechus* is not known to vector pathogens or other associated organisms [18].

We hereby report the establishment of the Tiger longicorn beetle, *Xylotrechus chinensis* (Chevrolat 1852) a new invasive alien longhorn beetle in Europe, specifically in Catalonia (NE Spain). The species was described from Shanghai (China) and is native to the East Palaearctic region (NE China, Taiwan, Korean peninsula, and Japan). Sometimes it is also called the Mulberry borer, although this name is more frequently used for *Apriona rugicollis* Chevrolat 1852 (Cerambycidae Lamiinae). In Japan, it is called ‘torahu-kamikiri’, and in France it is ‘perceur chinois’. There is no common name in Spanish and Catalan languages as yet, and we propose ‘Escarabajo-avispa taladro de las moreras’ and ‘Escarabat-vespa barrinador de les moreres’ respectively, since, as in other species of Clytini beetles, *X. chinensis* is a wasp-mimicking longhorn beetle.

The first record of the Tiger longicorn beetle in Europe was an interception in Weissenhorn, a town in the district of Neu-Ulm in Bavaria (Germany). Two specimens (one male and one female) emerged on the 15 and 19 June 2007 from a wooden packaging box from China, which had been marked as having been treated with methyl bromide. The beetles lived for 3–4 weeks in quarantine [19].

*X. chinensis* is an economically important pest whose xylophagous larvae feed mainly on mulberry trees (Moraceae) like the White mulberry, *Morus alba* Linnaeus 1753 and the Korean or Chinese mulberry, *Morus australis* Poiret 1797. However, larvae have also been recorded from apple, *Malus pumila* Miller 1768, pear *Pyrus* sp., (Rosaceae), and common grape (Vitaceae), *Vitis vinifera* Linnaeus 1753, all of them having great economic importance in southern Europe (see [20] for literature review of host plants). In Catalonia, 11,234 Ha are used for apple production, 7778 Ha for pear production and 55,133 Ha for vineyards, the latter being only 5.69% of the Spanish total [21].

It is likely *X. chinensis* will spread in Europe. The damage to mulberry trees is already significant, as well as the economic consequences. Human safety in public parks with mulberry trees is also a concern since heavy beetle infestation increases the risk of falling branches. Indeed, this has already happened in one town in which *X. chinensis* has established, fortunately with no human injuries.

In this study, we delimited the current area invaded by this beetle in Catalonia, assessed their damage to mulberry trees, and tested the suitability of grape vines as an alternative host in laboratory studies. We also provide significant new data on the life history of the beetle, especially its behaviour, seasonality and reproductive capacity, determine how it kills mulberry trees so quickly, and suggest control methods for hindering its spread.

## 2. Materials and Methods

### 2.1. Insects in the Wild

About three visits per year (in May, July and October) were made to the towns of Cerdanyola del Vallès and Badia del Vallès during the period 2015–2017 checking for the presence of infested mulberry trees. We concentrated our efforts on the town of Barberà del Vallès, especially during the years 2016 and 2017, counting and mapping all public mulberry trees, and determining the number of those infested by *X. chinensis* larvae. We considered a tree to be infested if there was at least one emergence hole on its trunk or branches made by an adult *X. chinensis*.

### 2.2. Insects in Semi-Field Conditions

Ten freshly cut trunks (bolts) of mulberry trees of different diameters (10 to 30 cm) and lengths (40 to 120 cm) with clear signs of infestation by *X. chinensis* were collected in late October 2015. Eight were picked from four felled trees kept at Badia del Vallès’ municipal enclosure, and two came from one felled tree at Barberà del Vallès. They were placed in semi-field conditions within a 1.20 × 1.50 × 2.10 m^3^ wire mesh cage, located in a nearby forest. In this cage, the beetle larvae these bolts contained were allowed to develop through the autumn and winter.

Next year, in 2016, from mid-June to mid-October, the cage was, with few exceptions, checked 2–3 times daily, in order to look for adult emergences and establish the beetle’s seasonality and the sex ratio. At the end of each day (20:00 h), all insects emerged were withdrawn from the cage, counted, sexed and either placed in insectaries (50 × 25 × 32 cm^3^) at laboratory temperature or refrigerated at 18 °C in an incubation chamber (ref. Eri-882/2, SCLAB, Barcelona, Spain) within labelled 500 cm^3^ plastic containers (ref. 20283, www.envasesindustriales.com, Guadalajara, Spain). The latter contained a layer of cellulose paper covering the bottom and walls so that the beetles could hide and rest easily, and to avoid overcrowding, each container held between one and six specimens. For a short period in 2016 (27 June to 10 July), adult emergences were not checked daily and the 10 beetles emerged within this period (4 males, 6 females) were allowed to stay in the cage until they were removed on July 10 at 20:00 h, as mentioned above.

These same bolts were kept in the cage through the rest of 2016. They now contained new larvae originating from eggs laid by the 2016 summer beetles. These larvae developed through the rest of summer, autumn and winter of 2016 and yielded adult beetles in summer 2017. These beetles were counted, sexed and withdrawn from the cage the same way as mentioned above for those of 2016. Therefore, two seasons (for adults) were obtained: those of 2016 and 2017.

### 2.3. Climatic Data and First Adult Emergences

To analyse if main climatic variables, namely the average daily relative humidity, average daily temperature, and cumulative daily precipitation, had a significant effect on the first emergence dates of adult beetles after the winter and spring period, the values for these variables recorded by an automatic weather station (PCC-Inspire III-7, EMA-WE, Servei Meteorològic de Catalunya) located close to the semi-field cage where we kept *X. chinensis* were retrieved from 1st May to 15th July of years 2016 and 2017 (Table S1).

### 2.4. Studies on Potential Alternative Hostplants (Common Grape Vine)

A simple experiment was set up to assess the capacity of *X. chinensis* to (1) lay eggs on a hostplant different to its usual host (mulberry) when this was not available, and (2) for the hatching larvae to accept and be able to feed on this hostplant. Since the common grape vine, *Vitis vinifera*, was one of the few alternative hostplants recorded for *X. chinensis* (see above), this was the plant chosen for the experiment.

Therefore, on 20 July 2017, an insectarium (50 × 25 × 32 cm^3^) was set up containing two freshly cut main arms of grape vine (50 cm, 5 cm diam.; 34 cm, 4 cm diam.) collected from a nearby vineyard. This same day, 6 males and 6 females were added to the insectarium. In the next days, until 5 August, up to 19 more beetles (11 males, 8 females) were added. And again, on 4 August 2017, a second insectarium was set up in a similar way. Here, three freshly cut main arms of grape vine (22 to 45 cm, 5 cm diam.) were added together with 26 beetles (13 males, 13 females). Beetles introduced in these insectaria came from those collected in the semi-field cage the same day, as well as from those kept in the chamber at 18 °C and collected in the cage less than two weeks prior. By 14 August, all beetles were dead in both insectaria.

In March 2018, under a binocular, we thoroughly removed the bark scales from the surface of the five arms of the grape vine kept in the insectaria, looking for egg remains (dead or hatched) and larval penetrations of *X. chinensis*. Later, using a scalpel, knife, and finally an electric woodsaw, we looked for overwintering larvae hidden within the vines’ arms, especially around the points where fallen frass (on the insectaria floors) had revealed the presence of larvae.

## 3. Results and Discussion

### 3.1. Arrival and Current Area Invaded in Catalonia

*X. chinensis* reached Catalonia no later than 2012, since based on information provided by citizens keeping mulberry trees in their gardens, first sightings of the beetle took place in July 2013. This is because it overwinters as larva within mulberries, pupates in spring, and the adults (those that the citizens saw) emerge mostly in July. When we first visited the area in October 2014, *X. chinensis* was already well established in the towns of Cerdanyola del Vallès, Badia del Vallès and Barberà del Vallès. We only learned about the presence of this pest in the town of Ripollet in February 2018 and visited it twice to date, concluding that *X. chinensis* had also invaded this municipality, most likely at around the same time as the other towns.

The invaded area is located 12–15 km to the NW of Barcelona city, within the district of Vallès Occidental (Barcelona province). It covers 44.1 km^2^ and is centered around the coordinates 41°30′57″ N, 2°07′28″ E. It is not known how it was introduced, although wooden packaging originating from China or Korea is the most likely pathway. Yet the other invasive pathways mentioned by Cocquempot & Lindelöw [5] cannot be ruled out.

In October 2014, the presence of this invasive species was formally communicated to the Spanish Ministerio de Agricultura y Pesca, Alimentación y Medio Ambiente (MAPAMA).

### 3.2. Description of Life Stages

**Figure 1:**
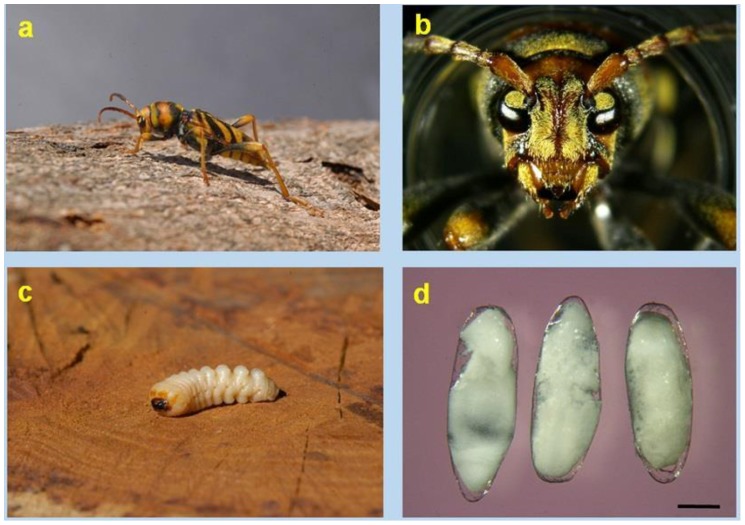
*Xylotrechus chinensis* life stages: (**a**) Female beetle walking on mulberry trunk; (**b**) Close-up of a beetle’s head, showing its widely separated antennae; (**c**) Last instar larva (extracted from its cavity); (**d**) Eggs (unfertilised), showing the oocyte mass protected by a transparent flexible chorion. Scale bar 0.5 mm. The pupa is not photographed. (Photos V. Sarto i Monteys).

*X. chinensis* body is quite large (15–25 mm in length, N = 25), the antennae are short and widely separated, characteristic bands adorn the elytra and the pronotum (Figure 1a,b). A complete set of physical characteristics for diagnostic purposes of the adult was provided by Cherepanov [6], including a B/W drawing of the beetle in dorsal view. Han and Lyu [20] also provide a description of the adult along with colour pictures showing it in dorsal and lateral views. Specific descriptions for the larvae, pupae and eggs seem to be lacking. The larvae look typically longhorn, i.e., they have a conical-shaped trunk with very well marked pseudopoda and a whitish colour (Figure 1c). The eggs are white, elongated, rounded at poles, narrowing towards the caudal end, with an average length of 2 mm, and width 0.7 mm. The oocyte is protected by a transparent flexible chorion with no apparent ornamentation when seen under a binocular stereomicroscope (Figure 1d).

### 3.3. Biology and Ecology

#### 3.3.1. Mating Behaviour and Egg Production Capacity

**Figure 2:**
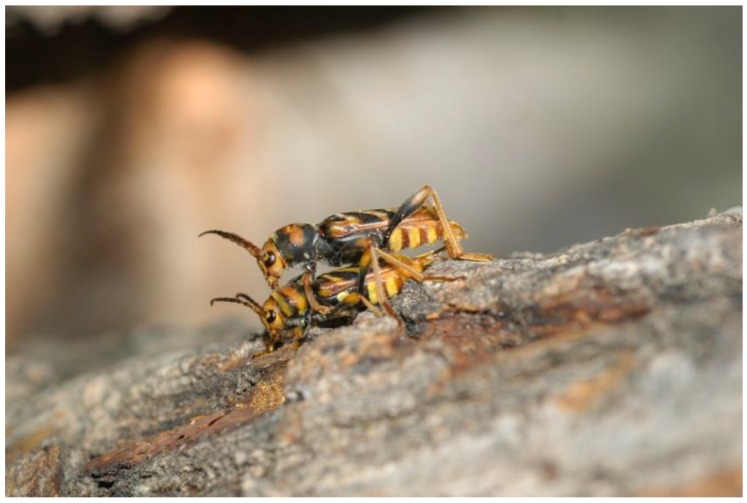
*X. chinensis* couple mating on a mulberry trunk. Male on top. (Photo V. Sarto i Monteys).

According to Iwabuchi et al. [22], in Japan, *X. chinensis* adults are active from 9:00 to 17:00. These authors described its mating behaviour in the wild. In sum, guided by the male pheromone, a female flies towards a male resting on a mulberry tree. Then passes within 1–2 m of the male, drops speed, circles and initiates a hovering flight near him. After that, the female alights on a leaf about 20–50 cm away from the male and starts walking towards the stem. When the female has approached to within 2–3 cm of the male, he immediately locates her, mounts and copulates with her (Figure 2). The copulation lasts more than five minutes. This behaviour is similar to that of the grape borer *X. pyrrhoderus* Bates 1873 [23].

Our observations were mostly limited to the specimens of *X. chinensis* seen within the big semi-field cage and the rather small insectaria mentioned above. This was not the ideal situation (see for instance [24]), but in many instances, it was the only possible approach. We noticed that, in the cage, all insects emerged in the morning (10:00 to 12:00 h) when the sun rays reached the trunk inside. After emergence, adults were immediately ready to mate; and mating took place on the mulberry trunks or on the cage walls. After mating, caged females were seen ovipositing on clefts of the bark of the same cut mulberry trunks, on their lower third, which was more humid. The beetles are relatively good flyers as well as wasp-mimickers. Indeed, they are Batesian mimics of aculeate Hymenoptera, such as species of *Vespula* and *Polistes*, conspicuously patterned yellow and black, often moving their body and antennae resembling a vespid (body quickly raised/lowered by the legs, antennae forward-facing, more or less parallel to each other, quickly moving up-and-down, not in unison). When threatened (for instance when they are caught), they produce a guttural sound clearly audible to humans, somewhat similar to a wasp buzz-like noise. Males are very active and aggressive sexually, and copulate with different females, although no specific counting was done. In one case a copula lasted for approximately 5 min. Males that had been refrigerated (18 °C) for three weeks recovered their sexual drive as soon as they were placed at 25 °C in the lab, actively seeking females for mating.

Ten *X. chinensis* virgin females were kept isolated in jars until they died; six of them had laid a few eggs which were counted. They were dissected and all eggs resting within their abdomens were counted. In all cases, abdomens were absolutely packed with eggs. Table 1 summarises the counts.

In our sample, females produced an average of 83.4 eggs, SD 9.02, although generally not all eggs were laid. We also dissected ten mated females which had been placed in the insectaria until they died. They all had an abdomen that was clearly deflated dorsally; the tegument on the dorsal side of the abdomen, protected by wings and elytra, was much less sclerotised than that of the ventral side. The eggs remaining in their abdomens were counted (Table 2), resulting in an average of 15.2 eggs, SD 10.61, i.e., around 18% of the total average egg production capacity mentioned above.

#### 3.3.2. Seasonality

*X. chinensis* is univoltine in Asia and adults emerge from July to August [25]. In Catalonia, we gathered data for two consecutive summers, 2016 and 2017, based on caged specimens kept in semi-field conditions (see above).

**Figure 3:**
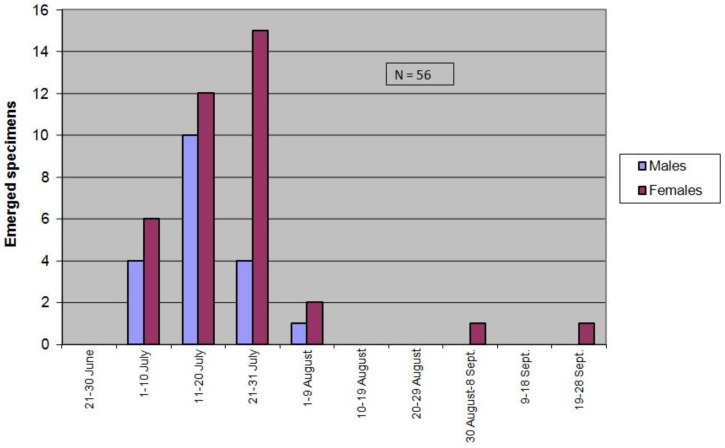
*X. chinensis* 2016 adult seasonality at Barberà del Vallès (Barcelona, Catalonia).

In the summer of 2016, only 56 adults (33.93% males; 66.07% females; male/female ratio: 0.51) emerged from mulberry bolts collected in October 2015 (see above). Emergences started 1 July and ended 3 August (91.07% in July; 5.36% in August). There was no clear protandry or protogyny. Exceptionally, two females emerged extemporaneously on 5 and 24 September (3.57%) (Figure 3).

**Figure 4:**
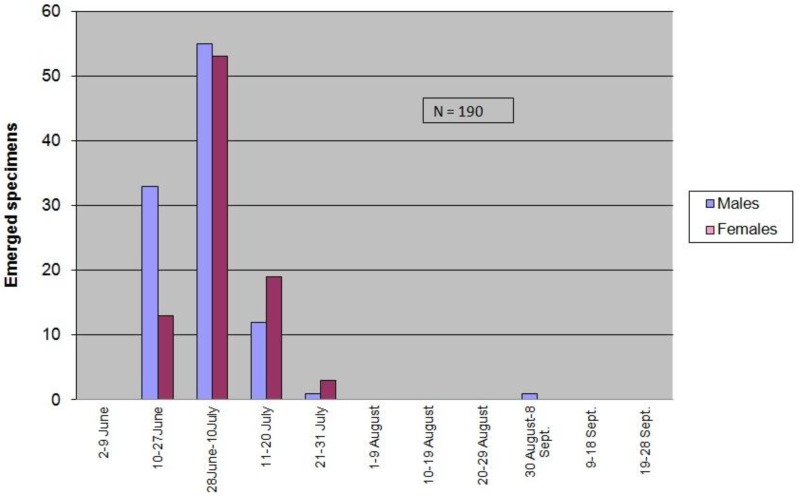
*X. chinensis* 2017 adult seasonality at Barberà del Vallès (Barcelona, Catalonia).

In the summer of 2017, a total of 190 adults emerged (from eggs laid by females that emerged in the summer of 2016). Specific figures were 102 males (53.68%), 88 females (46.32%), giving a male/female ratio of 1.16; quite different to that of the previous summer. Indeed, protandry became clear and males were slightly more abundant than females. Emergences started 15 June and ended 27 July (34.74% in June; 64.74% in July). Again, exceptionally, one male emerged on 31 August (0.53%) (Figure 4).

Therefore, as in NE Asia, *X. chinensis* was univoltine in Catalonia, with July being the main month for the adults to be found. Very few adults emerged in August. Sustained high temperatures in June though may advance emergences to the middle of this month, as happened in 2017. Using the climatic data mentioned above, correlations could not be established concerning relative humidity and precipitation, but average daily temperatures seemed to be important. Indeed, in 2016, the first adult emergence (1 July) occurred after ten consecutive days with an average daily temperature above 20 °C; in 2017, the first adult emergence (15 June) occurred after seven consecutive days of these same values.

It is known that some cerambycids can take 1 or 2 years to develop and emerge as adults from the same bolt of host material in the same conditions, i.e., some larvae will undergo prolonged diapause (see for instance [26]). There are no records of *X. chinensis* taking more than one year to develop if in live trees. In late 2016, we purposely did not dissect bolt subsamples looking for larvae, but we checked five pieces of bark which had been removed accidentally while moving the bolts. The latter only showed underneath about the same size larvae, which appeared to have emerged in July 2016, i.e., we could not see any mature larva indicative of being in prolonged diapause from the 2015 generation. Yet, the possibility that some individuals may have taken more than one year to develop to adults cannot be ruled out.

### 3.4. Larval Development and Damage to Mulberries in Catalonia

According to Orlinski [17], damage by many *Xylotrechus* species is limited to dead or injured broadleaf trees or conifers, but some species, like the Altai larch longhorn beetle *X. altaicus* (Gebler 1836), can attack and kill living trees. The latter applies to *X. chinensis*. Indeed, in Catalonia, this beetle’s larvae have been observed boring into the phloem and xylem tissues of medium and large (old) mulberry trees, which are still alive and healthy. Both Catalan mulberries, *Morus alba* and *M. nigra* Linnaeus 1753, were attacked. Notwithstanding the above we have observed they can also feed successfully on cut mulberry logs.

White mulberries (*Morus alba*) are planted extensively in Catalonia, mostly for landscaping, as ornamental and shadow trees. Since fruits are abundant and fall on the floor dirtying it, many towns use a fruitless variety where shade is desired without the fruit. Black mulberries (*Morus nigra*) are also used, although less than White ones [27]. Therefore, mulberries are common in recreational and playground parks, as well as lining streets and avenues in many Catalan towns, not to mention in many private gardens. Economic losses result from the attack and associated mortality of healthy trees, which have to be pruned for safety or removed and destroyed.

In February 2016, the town of Barberà del Vallès had 506 mulberry trees spread over its public avenues, streets and squares. Mulberries could also be found in private gardens though these were not counted. Among the 506, 82 (16.21%) were infested by *X. chinensis*, 16 of which were in such poor condition that local authorities decided to cut and replace them with new ones. Figures were worse for the towns of Badia del Vallès and Ripollet, with 233 and 356 public mulberry trees respectively, the former with 32.62% of trees infested, the latter with 44.84% (as to October 2017). The town of Cerdanyola del Vallès had 530 public mulberry trees, with about 10% infested according to the municipal gardening services (as to March 2018).

One of these mulberry trees received a special follow-up. In the summer of 2015, when it must have been infested by *X. chinensis* for the first time, it showed no emergence holes. Larvae developed hidden under the bark and overwintered in the tree; and an additional inspection carried out in February 2016 showed, as expected, no emergence holes. However, the following summer (inspection 21 July 2016) revealed 27 emergence holes, to which only one would be added during the next two weeks. These 28 holes were tagged (painted orange-red). The following summer, 15 new holes were counted on this mulberry (inspection 11 August 2017).

**Figure 5:**
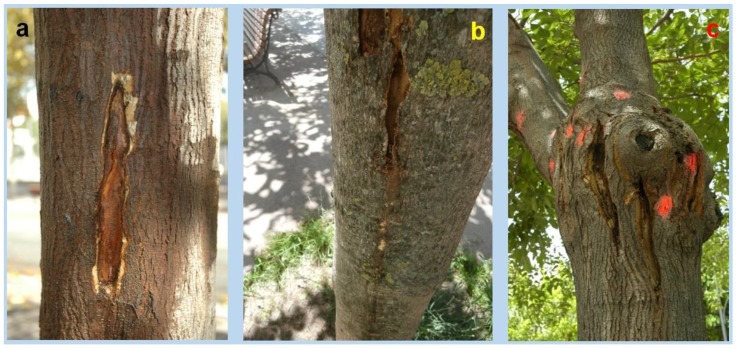
Exposed larval feeding cavities of *X. chinensis* in mulberry phloem: (**a**,**b**) along trunks; (**c**) at the base of main tree branches, red/orange dots point out adult emergence holes ((**a**,**c**): photos V. Sarto i Monteys; (**b**): photo G. Torras).

Based on our observations with live mulberry trees in parks and gardens, *X. chinensis* larvae feed hidden underneath the periderm (formed by cork and cork cambium) of mulberry trees, either along trunks (Figure 5a,b) or at the base of main tree branches, where they join the trunk (Figure 5c). Mature or old trees are preferred; attacks on young trees are rare, most likely because their narrower trunk diameter provides less food and shelter to the larvae. The latter feed only on the phloem and vascular cambium, tunneling upwards or downwards, and so making longitudinal cavities that reach 15–25 cm in grown larvae.

**Figure 6:**
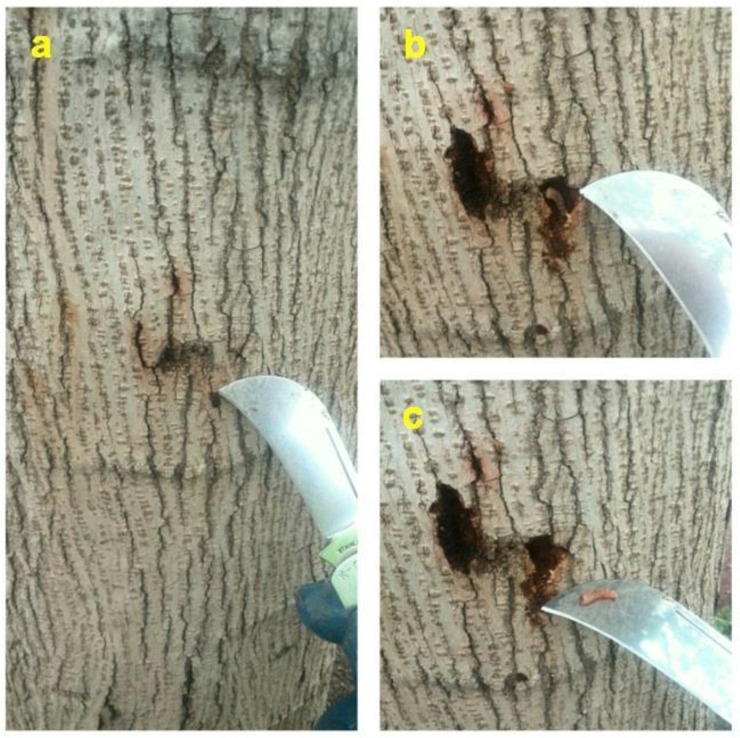
*X. chinensis* larva within its feeding cavity in the phloem of a mulberry tree trunk. The sequential images (**a**–**c**) show the process of exposing such larval cavities. Note the tiny frass protrusions through slits and cracks on the periderm (bark) in image (**a**) hardly reveal the presence of the beetle larvae underneath. (Photos Jordi Serra).

**Figure 7:**
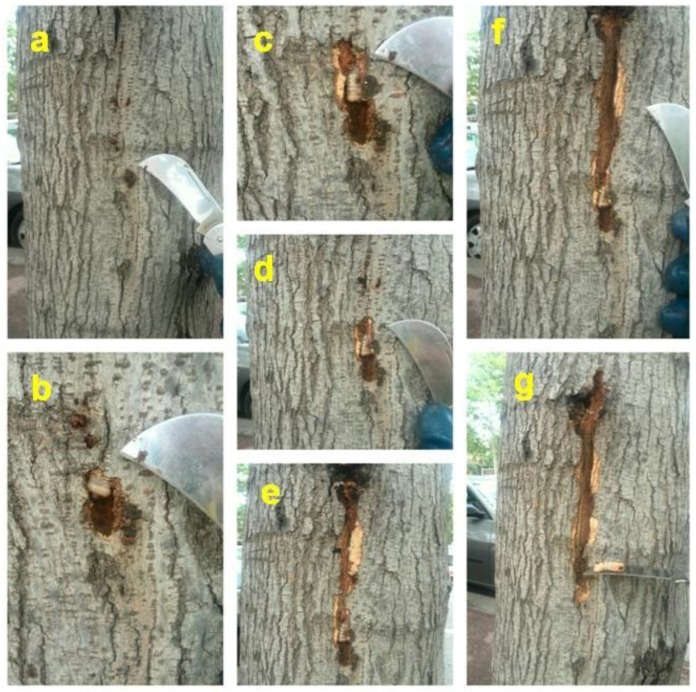
Same as in Figure 6. The sequential images here go from (**a**–**g**). (Photos Jordi Serra).

**Figure 8:**
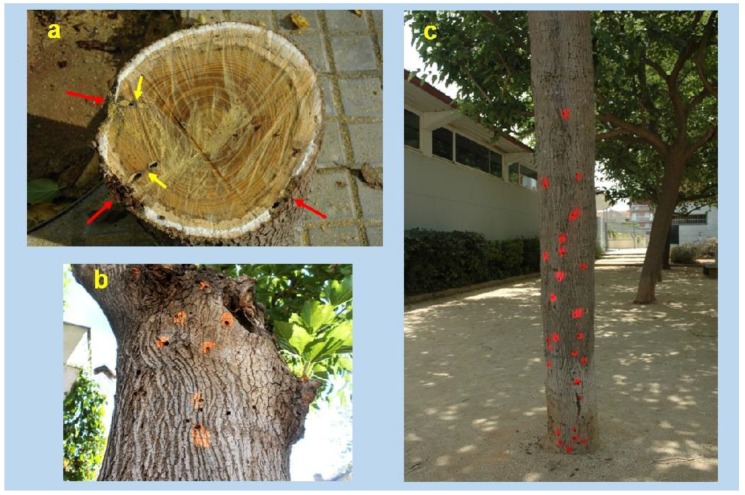
(**a**) *X. chinensis* phloem and xylem larval cavities. Note white latex exuding in mulberry phloem after cutting the trunk, excepting the sections consumed and damaged by the beetle’s larvae (red arrows). Xylem tunnels (yellow arrows) are excavated generally at right angles with the phloem cavities; the larvae will pupate in them. (**b**,**c**) Adult emergence holes in heavily infested mulberry trees. ((**a**,**c**): photos V. Sarto i Monteys; (**b**): photo G. Torras).

Aruga [28] had already mentioned that the larvae of *X. chinensis* feed mainly on ‘cambium’; we interpret this author’s ‘cambium’ as equal to phloem plus vascular cambium as mentioned above. In mulberry trees, especially in those belonging to the fruitless variety, the periderm is quite thin and dries out when the underlying phloem is consumed, so small cracks or slits, but not proper holes made by the larvae, appear on its surface which are used for aeration and some removal of frass. Most frass though stays within the cavities. These tiny periderm openings, not being holes, are not very obvious while the larva feeds and hides underneath, making larval detection difficult (Figure 6 and Figure 7). Interestingly, mulberry phloem contains a white latex (see Figure 8a) which is usually exuded after tissue injury and serves mainly as defense against herbivorous insects; this latex does not appear to prevent *X. chinensis* larvae from eating the phloem.

When around mid-May the larvae have finish their development, they tunnel a new cavity through the xylem towards the trunk core, reaching both sapwood and hardwood (Figure 8a, yellow arrows). This xylem cavity is generally at a right angle with the longitudinal (phloem) cavity, and will be used as a pupation chamber. When the adult emerges, it goes to the outside and makes a well-defined round emergence hole, with a diameter similar to that of the beetle’s mesothorax, its widest body section (generally 5–6 mm). Therefore the number of holes that appear on the bark of mulberries is a measure of the trees’ degree of past infestation (Figure 8b,c).

Nearly all emergence holes appear on trunk areas facing direct morning sunlight, i.e., on the warmer side of the tree. In highly infested trees, holes distribute all along the trunk, from bottom to top, and also over the base of main tree branches, where the latter joins the trunk. When trees are moderately infested, holes tend to concentrate on the mid-upper trunk and base of main branches.

**Figure 9:**
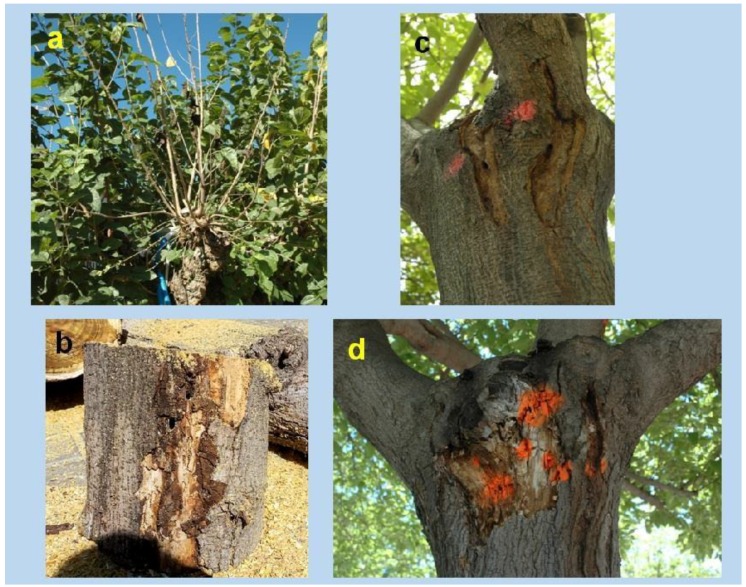
Damage to mulberries by *X. chinensis* larvae: (**a**) Loss of leaves in one of the main branches (trunk stump) of a mulberry tree due to phloem sap stoppage originated by *X. chinensis* larval damage at the stump base. (**b**) Slice of mulberry trunk with detached periderm showing consumed and dead phloem, as well as the remains of masses of packed frass and some adult emergence holes. (**c**) Similar to (**b**) but at the base of a main branch, frass already gone. (**d**) Exposed xylem sapwood beginning to dry and crack after heavy damage by *X. chinensis* larvae. (**a**,**c**,**d**: photos V. Sarto i Monteys; **b**: photo G. Torras).

Damage to mulberries is mostly produced as a result of the progressive disappearance of the phloem and vascular cambium due to larval feeding (Figure 8a, red arrows). The room originally taken up by these living tissues is replaced by a cavity along which the larva moves up and down and packs larval frass. Therefore, in damaged parts, the two-way flow of water and food (phloem sap) stops. When this happens at the base of big branches, shoots start to wilt and leaves are lost (Figure 9a). In severe infestations, when several cavities meet, the periderm covering them easily breaks off showing the woody xylem underneath (Figure 9b,c); without cover, the xylem sapwood begins to dry and crack externally, this being especially obvious at the base of big branches (Figure 9d). Cracks in damaged bark and emergence holes that reach deep into the xylem may also facilitate infections by bacteria and fungi, as well as infestations by other insects. Eventually this causes the death of the tree.

*X. chinensis* females may also lay eggs on cut mulberry trunks. Their larvae will feed on the dead phloem and vascular cambium, as described above for live mulberries. Indeed, infested trunks of mulberry trees cut and collected in late October 2015, and placed in a semi-field cage (see above), produced 56 healthy adults in the summer of 2016. After mating, caged females oviposited on the same mulberry trunks. These eggs produced another generation of larvae resulting in 190 new adults in the summer of 2017. During development, larvae produced frass, which mostly stayed in the cavities; a part of it though was expelled outside the bolts and was visible accumulating on the cage floor. A rasping sound produced by the wood-feeding larvae could be clearly heard inside the cage from approximately early August to late October.

### 3.5. Host Plants Other Than Mulberries

*X. chinensis* larvae generally feed on mulberries, but apple, pear and common grape vine have also been recorded as possible host plants [20]. As far as we know, there have been no sightings of this beetle in nearby orchards affecting any of these hosts but it is probably too early to draw conclusions since the pest has only recently settled and garden caregivers and fruit growers are not yet familiar with it.

In the laboratory, we offered five freshly cut main arms of common grape vine to *X. chinensis* females (see details above). By the end of August 2017, four spots of fine frass could already be seen accumulating on the insectaria floors under the vines’ arms (one in the insectarium set on 20 July, three in the insectarium set on 4 August), suggesting some larvae might have survived on that host from eggs laid by *X. chinensis* females. However, we expected many more frass spots, given the number of females introduced in both insectaria. In March 2018, the vine arms were opened (see Methods) and we found inside four healthy overwintering cerambycid larvae, although they were not *X. chinensis* but another species (most likely *Xylotrechus arvicola*) whose eggs or young larvae must already have been in the grape vines collected in the wild in summer to run the experiments. In addition, we could not find any remains of *X. chinensis* eggs (dead or hatched) on the surface of those five grape vine arms. We do not know why *X. chinensis* females did not oviposit on the vines. Certainly, the diameter of the vines offered in the experiment was only of ca. 5 cm, probably too thin to house *X. chinensis* larvae, and we do know that young mulberries (diameters of up to ca. 15 cm) are generally ignored by *X. chinensis* females. In sum, we conclude that, at least in our laboratory conditions and with the vines used, *X. chinensis* did not use common grape vine as an alternative hostplant. Further research should be carried out to go deeper into these issues.

There is an example of a native *Xylotrechus* (namely *X. arvicola*), whose larvae are polyphagous on different wooden cultivars and wild deciduous trees, accepting common grape vine as a new hostplant. Its incidence was more frequent in those plots situated in the vicinity of rivers, suggesting the beetles accessed the grape vines from their common hosts that grow on river banks [16]. It is possible something similar might happen with *X. chinensis*, i.e., the colonization of apple and pear trees, and perhaps thicker grape vines, from nearby mulberries.

### 3.6. Pathway and Vector Capacity of Pathogens

We still do not know what was the pathway that brought *X. chinensis* to Catalonia and have not observed this beetle vectoring any pathogen.

### 3.7. Possible Control Measures and Suggestions for Future Studies

**Figure 10:**
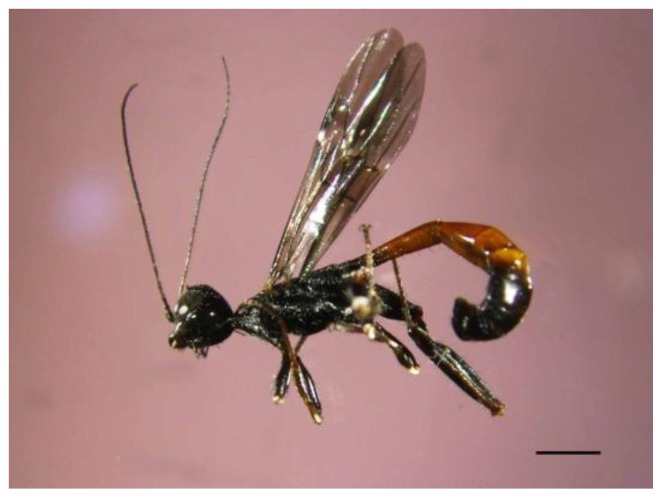
A native stephanid wasp, *Stephanus serrator* (a male), a likely parasitoid of *X. chinensis* larvae (see text for details). Scale bar 2 mm. (Photo V. Sarto i Monteys).

Aruga [28] listed three control measures for *X. chinensis*, although they are simple and applicable to most coleopteran and lepidopteran tree borers. They are as follows: (1) Adults are caught and killed; (2) if frass protrusions reveal the presence of larval cavities, a suitable insecticide should be injected into these cavities; and (3) larvae can be killed by inserting metal wire inside the cavities. We believe Aruga’s methods are not adequate to control this pest since they are practically unworkable. Possible control methods might be as follows:

(a) Physical control. Removal and destruction (burning or grinding) of heavily infested mulberry trees. To date this has been the only measure used in the invaded towns.
(b) Chemical control. To date no insecticides have been tested for their efficacy against *X. chinensis* in Spain. However, to our understanding, two approaches are feasible:
  (1) Targeting ovipositing females, their eggs and first instar larvae. Since females lay eggs on mulberry barks (trunks and main branches) from mid-June to mid-August, spraying contact insecticide on such barks at the beginning of June should suffice to protect the trees. This assumes a two-month half-life under outdoor conditions of the insecticide used; if shorter, a second treatment might be carried out in mid-July. One such insecticide might be chlorpyrifos, sold under many brand names and registered and authorized in Spain for the protection of agricultural crops. It is an organophosphate acting as a contact insecticide and stomach poison, considered moderately hazardous to humans by the World Health Organization. It worked quite well against the Castniid Palm Borer, *Paysandisia archon* (Burmeister), an exotic invasive moth in Europe that attacks palm trees [29]. However, special regulations applying to insecticide use in public areas may hinder its implementation.
  (2) Targeting larvae (all instars) feeding in the tree phloem. This would require the use of a systemic insecticide, for instance abamectin, mostly a stomach poison, introduced into the xylem through endotherapy, i.e., the trunk injection of a sap-compatible solution of the insecticide. The xylem sap moves upwards through the vessels and the active ingredient is so transported to the phloem. This confines the applied insecticide only within the target tree, thereby making it particularly useful in urban situations. Abamectin has shown good results against bark beetles (see for instance [30]) and will be used experimentally in the town of Barberà del Vallès in the spring of 2018.
  Injecting insecticide into the emergence holes seen on mulberry barks, apart from being very time-consuming, would not be efficient since they are not really well connected to the phloem cavities inhabited by larvae. Obviously, an emergence hole leads to an empty tunnel where the larva pupated and the hole is only made when the adult abandons the tree.
(c) Pheromone control. Since the three components of the male-produced aggregation pheromone of *X. chinensis* are known, namely 2, 3-octanediol, 2-hydroxy-3-octanone and 3-hydroxy-2-octanone [22,25], studies to test their efficacy for mass-trapping and/or mating disruption might be worth-while.
(d) Biological control. During the course of this work we discovered the native stephanid wasp *Stephanus serrator* (Fabricius 1798) (Figure 10) as a likely parasitoid of *X. chinensis* larvae. Since it was not possible to find the corpse of the parasitized beetle larva within the several bolts infested by *X. chinensis*, we only can assume the latter served as the host for *S. serrator*. This assumption is reinforced by the fact that local woodborers do not generally appear as pests of mulberries in Spain [31] and especially because no bark beetles or woodboring beetles other than *X. chinensis* emerged from our bolts.

The genus *Stephanus* Jurine, 1801, comprises five species worldwide. *S. serrator* is the only one inhabiting the Western Palaearctic, two occur in the Eastern Palaearctic and the other two are in the Oriental region [32]. In Spain, *S. serrator* has been scarcely reported although it is likely distributed all over Iberia [33].

Stephanids are solitary idiobiont endoparasitoids of xylophagous coleoptera, mainly Cerambycidae and Buprestidae, but there are records of attacks on other coleopteran families, as well as on Hymenoptera Siricidae and solitary bees [34]. Specifically, *S. serrator* has been reported as a parasitoid of xylophagous coleoptera of conifers and fruit trees [35]. At least in one case in Valencia, Spain, Selfa et al. [33] mentioned one *S. serrator* wasp emerging from a *Xylotrechus arvicola* larva that was boring in hazelnut, *Corylus avellana* Linnaeus 1753.

Only one *S. serrator* wasp was seen (on 30 July 2016) in the experimental cage. The latter contained bolts cut from living trees that had been infested by *X. chinensis* larvae the previous summer of 2015. Therefore, if the assumption above is correct, a female mother wasp must have parasitized in the wild one *X. chinensis* (mid or late instar) larva most likely in September 2015, since those trees were cut in October and housed (some bolts) in the cage where the descendant wasp was found next July 2016. Since in summer 2016, 56 adult beetles and one parasitic wasp emerged from the bolts, i.e. a parasitic ratio of only 1.75%, this might suggest this wasp species is starting to recognize this new invasive beetle as a potential host, and studies aimed at analyzing its potential as a biological control agent could be performed.

## 4. Conclusions

We have revealed the invasion of a new alien beetle species to Europe, the longhorn *Xylotrechus chinensis*, originating from NE Asia. It has settled in Catalonia (Spain), occupying at present an area of at least 44.1 km^2^, where it has been shown to severely infest (ca. 10 to 45%) and eventually kill mulberry trees in private and public grounds. We have provided new significant insights into its life history, seasonality and reproductive capacity and documented the type of damage caused to mulberries; the latter has been thoroughly described to facilitate inspection by others. Based on all data gathered, we have suggested possible control methods to hinder its spread. We have discovered a native stephanid wasp, *Stephanus serrator*, as a likely parasitoid of *X. chinensis* larvae, which might be tested as a biological control agent. We have shown that, at least in our laboratory conditions and with the vines offered to the females, *X. chinensis* has not used common grape vine as an alternative hostplant. Both plants, mulberries and grape vines, are important in Catalonia and Spain, the former providing shade and ornament to many streets and avenues, the latter having great economic significance in the Mediterranean wine production areas. We believe the likelihood of this beetle widely spreading in Europe is very real.

## Figures and Tables

**Table 1:** Egg production capacity of ten virgin *Xylotrechus chinensis* females.

Virgin Females	#1	#2	#3	#4	#5	#6	#7	#8	#9	#10
Eggs laid	0	19	4	6	0	0	5	13	7	0
Eggs remaining in the abdomen	77	69	98	67	77	84	89	69	74	76
Total eggs	77	88	102	73	77	84	94	82	81	76

**Table 2:** Eggs remaining in the abdomens of ten mated *Xylotrechus chinensis* females which had been placed in insectaria until they died.

Dead Mated Females	#1	#2	#3	#4	#5	#6	#7	#8	#9	#10
Eggs remaining in the abdomen	17	19	0	28	0	23	21	10	6	28

## Supplementary Materials

The following are available online at http://www.mdpi.com/2075-4450/9/2/52/s1, Table S1: Climatic data (average daily relative humidity, average daily temperature, and cumulative daily precipitation) recorded by the automatic weather station of Vilanova del Vallès, located close (8 km) to the semi-field cage where the experiments were run.
